# Using Peek as a Framework Material for Maxillofacial Silicone Prosthesis: An In Vitro Study

**DOI:** 10.3390/polym15122694

**Published:** 2023-06-15

**Authors:** Pinar Cevik, Arzu Zeynep Yildirim, Emine Hulya Demir Sevinc, Aykut Gonder, Sudarat Kiat-Amnuay

**Affiliations:** 1 Department of Prosthodontics, Faculty of Dentistry, Gazi University, Ankara 06490, Turkey; 2 Department of General Practice and Dental Public Health, School of Dentistry, The University of Texas Health Science Center at Houston, Houston, TX 77054, USA; 3 Houston Center of Biomaterials and Biomimetics, Houston, TX 77054, USA

**Keywords:** acrylic resins, materials testing, maxillofacial prosthesis, polyetheretherketone, tensile strength, silicone elastomers

## Abstract

There are often bonding problems between acrylic resins and silicone. PEEK (polyetheretherketone), which is a high-performance polymer, has great potential for the implant, and fixed or removable prosthodontics. The aim of this study was to evaluate the effect of different surface treatments on PEEK to be bonded to maxillofacial silicone elastomers. A total of 48 specimens were fabricated from either PEEK or PMMA (Polymethylmethacrylate) (n = 8). PMMA specimens acted as a positive control group. PEEK specimens were divided into five study groups as surface treatments as control PEEK, silica-coating, plasma etching, grinding, or nano-second fiber laser. Surface topographies were evaluated by scanning electron microscopy (SEM). A platinum-primer was used on top of all specimens including control groups prior to silicone polymerization. The peel bond strength of the specimens to a platinum-type silicone elastomer was tested at a cross-head speed of 5 mm/min. The data were statistically analyzed (α = 0.05). The control PEEK group showed the highest bond strength (*p* < 0.05) among the groups. No statistical difference was found between control PEEK, grinding, or plasma etching groups (*p* > 0.05). The lowest bond strength was seen in the laser group, which was not statistically different from silica-coating (*p* > 0.05), and statistically different from control PEEK, grinding, or plasma groups (*p* < 0.05). Positive control PMMA specimens had statistically lower bond strength than either control PEEK or plasma etching groups (*p* < 0.05). All specimens exhibited adhesive failure after a peel test. The study results indicate that PEEK could serve as a potential alternative substructure for implant-retained silicone prostheses.

## 1. Introduction

Silicones are frequently used in maxillofacial prosthesis fabrication due to their acceptable physical and mechanical properties [1]. The retention of silicone prostheses is generally provided by implant systems in the maxillofacial region and acrylic resins are mostly used as a substructure between the implant and the silicone [2,3]. However, there are often bonding problems between acrylic resins and silicone [4]. It needs to be renewed due to the breaking of the bonding between silicone and the infrastructure, or the fractures and/or cracks in the acrylic resin [2,5,6]. It is thought that the bonding problems between silicone and acrylic resins used in maxillofacial prostheses are due to the chemical structure difference between the two materials [7,8,9]. Adhesive systems such as primers are applied on acrylic resin in an attempt to eliminate weakness in the bond between them. It has been reported that there is no bonding between the two materials with their different compositions when the primer is not applied [8]. Therefore, adhesive primers are used to enhance the bond between acrylic resins and facial silicone [10].

Primers have been stated to act as an organic solvent agent to react with the silicone and resin materials [11,12], and as a chemical intermediate component of the silicone and the acrylic resin substrates. The hydrophilic and hydrophobic groups of primers react with the functional groups of silica [13]. At the same time, the activation of primers directly on the substrate surface increases the wettability of the substrate and consequently impregnates the surface layer with the polymer content [14]; they act as chemical intermediates of silicone and acrylic resin. Furthermore, it has also been reported that they activate the surfaces via etching or promoting hydrogen bonding and covalent coupling. Some other studies have also suggested the use of primer to increase the bond between silicone and acrylic resin [5,15].

PEEK was introduced as a dental material in 1992 and was initially used for aesthetic abutments due to its superior color compared to metals [16,17]. PEEK is resistant to chewing forces, cost-effective, and anti-allergic. It has several advantages over titanium, such as providing an aesthetic appearance in thin biotype mucosa, good dimensional stability, and an elastic modulus between cortical and cancellous bone [18,19,20,21,22]. Therefore, PEEK is a suitable alternative to traditional implant materials such as titanium and stainless steel and is used in many dental and prosthetic treatments, including fixed restorations, individual abutments, dental implants, removable prostheses, and prosthetic infrastructures [23,24,25].

PEEK needs to be subjected to different surface treatments in order to increase its bonding to polymers [26]. Sandblasting and silica coating [27,28], plasma applications [29,30], different laser systems [29,30,31], sanding and mechanical abrasion [32], and different acid applications [29,30,31,32] are the surface treatments that can be applied to PEEK. To the best of our knowledge, there is no study evaluating the bond strength between silicone and PEEK in the field of maxillofacial prosthodontics. It is important as it is the first study to be done on this subject. The bonding of PEEK with silicone and potential surface treatments are subjects of interest of this study. Conventional applications include the acrylic specimens to be used as a framework material for a maxillofacial silicone prosthesis. Therefore, the aim of this study was to investigate the feasibility of utilizing a new generation high-performance PEEK as a substructure between implant and silicone in maxillofacial prostheses. As a positive control group, polymethyl methacrylate (PMMA) acrylic specimens were included in this study. Additionally, this study aimed to examine the effect of various surface treatments on PEEK specimens to be bonded to maxillofacial silicone, which is a clinically relevant aspect. The null hypotheses of this study are: (1) There would be no differences among different surface treatments on PEEK to be bonded to one type of silicone elastomer with regard to the peel bond test; (2) There would be no difference between untreated PEEK specimens and untreated PMMA specimens.

## 2. Materials and Methods

PEEK rectangular specimens with the dimension of 60 mm × 10 mm × 3 mm were fabricated by milling from a high-grade, industrially manufactured PEEK disc in natural color (JUVORA™, JUVORA™ Ltd., Thornton-Cleveleys, UK). A total of 48 specimens (40 specimens were PEEK, 8 specimens were PMMA) prepared and divided into 6 (n = 8) groups according to the surface treatments applied to PEEK or PMMA. The surfaces of all the specimens were cleaned with acetone by using cotton pellets. To ensure the standard, 800-grit sandpaper was first applied to them.

The study groups are as follows:

Group Control (n = 8): It was the control group and a platinum primer (G611) (G611 Platinum Primer, Technovent Ltd., Bridgend, UK) was applied to the top of the PEEK specimens. One-coat primer was applied to the dried specimens with the help of a cotton pellet. The primer was left to dry without any further treatment.

Group Silica-coating (n = 8): Cleaned and dried PEEK specimens’ surfaces were silica coated with 30 µm silica-coated Al2O3 with Cojet Sand (3M ESPE, St. Paul, MN, USA). Sand particles were applied to the specimens for 10 s from a distance of 10 mm at a pressure of 2.5 bar.

Group Plasma (n = 8): Plasma etching was applied to the cleaned and dried PEEK surfaces. Oxygen plasma was applied under 150 m Tor at 100 W power [33]. An apparatus (Vakuum Praha, Prague, Czech Republic) with 13.56 MHz frequency power was attached to oxygen gas reactor. Oxygen gas was released at a volumetric flow rate of 30 sccm (standard cubic centimeters per minute) for 5 min to the top surface of each specimen. The oxygen plasma etching used for 1 min [34]. The specimens were kept under vacuum for gas stabilization.

Group Sandpaper (n = 8): The cleaned and dried specimens underwent sanding using 600 silicon carbide sandpaper following the initial application of 800-grit sandpaper. Subsequently, the specimens were cleaned and dried again.

Group Laser (n = 8): Nano second fiber laser (FiberLAST A.Ş., Ankara, Türkiye) was applied to the cleaned and dried PEEK specimens at a power of 2 W, a speed of 500 mm/s, and a frequency of 40 kHz.

Group PMMA (n = 8): Autopolymerized clear PMMA acrylic specimens (IMICRYL, Konya, Turkiye), with the dimensions of 60 mm × 10 mm × 3 mm were prepared, and used as the positive control group. A platinum primer (G611, Technovent Ltd., Bridgend, UK) was used on the surface prior to the silicone polymerization.

Surface topography of specimens from different groups was analyzed using scanning electron microscopy (SEM). In the control group, the surface topography was analyzed before the primer application, while in the other study groups, it was analyzed immediately after the surface treatment.

The surface roughness values of PEEK specimens were measured with a digital profilometer (Mitotoyo Surf Test SJ 201 P/M; Mitotoyo Corp, Takatsuku, Japan) following the surface treatments, including the control group. A constant force of 0.4 gf at 4 mm was applied to the specimen surface, and the obtained values were digitally generated on the front of the profilometer and recorded as Ra values.

**Figure 1:**
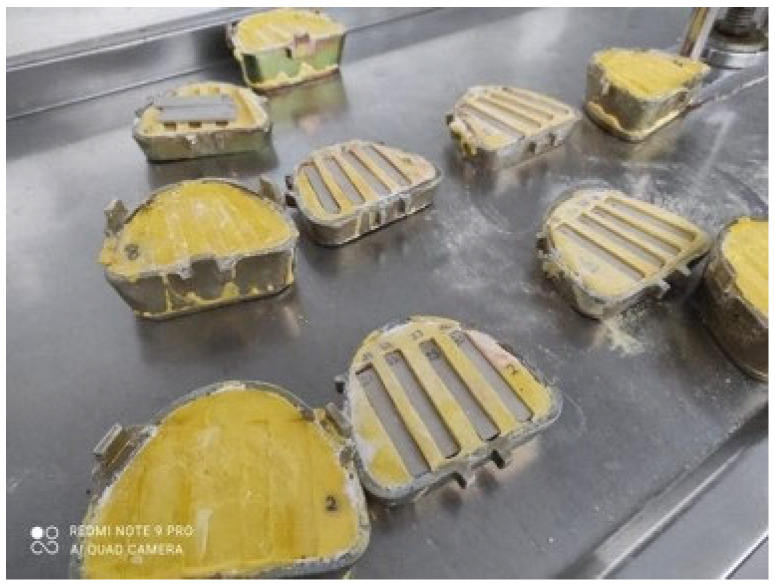
PEEK blanks to be bonded to silicone material.

Prior to silicone bonding, all the surfaces were cleaned with acetone, and dried. A high-temperature vulcanized (HTV) platinum-type maxillofacial silicone material (M511 Technovent Ltd., Bridgend, UK) was used in this study. First, PEEK blanks were inserted into the metal gypsum molds (as shown in Figure 1).

**Figure 2:**
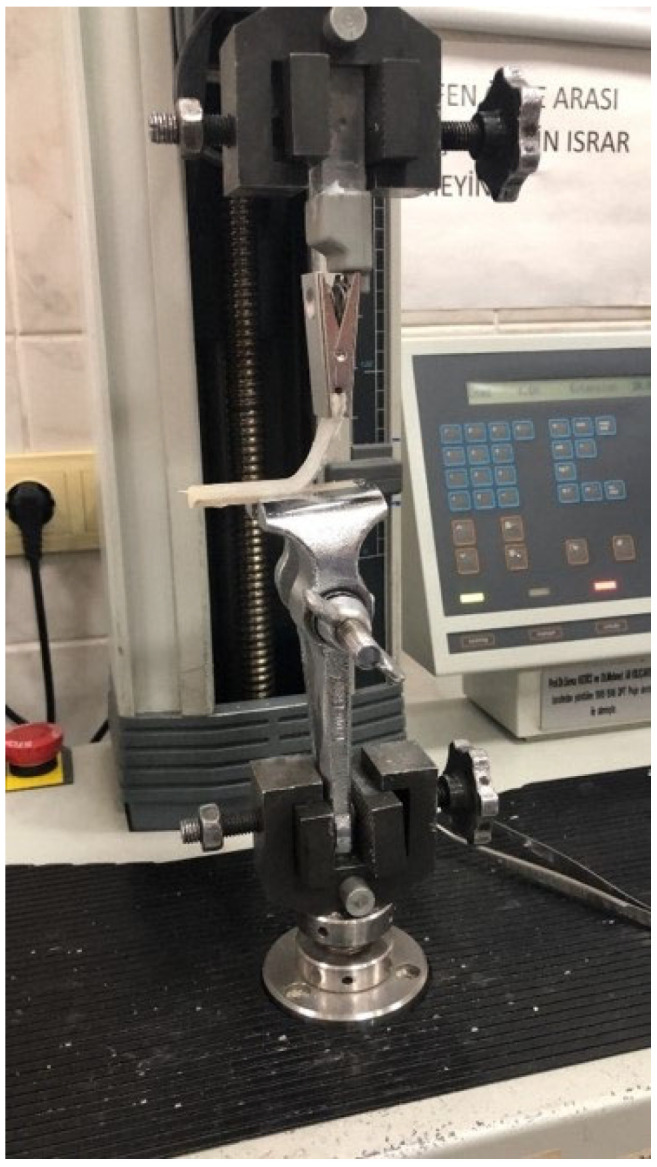
Application of 90-degree Peel test with an Instron machine.

The silicone material was prepared by mixing part A and part B in a 10:1 ratio, then packed onto a specified area of PEEK blanks. The silicone was polymerized by heating it in a dry-hot air oven at 135 C for 15 min. After polymerization, a peel test was performed to measure the bond strength between the PEEK and silicone. The 90-degree (90°) peel test was conducted using an Instron machine at a speed of 5 mm/min (Figure 2).

Types of failures were classified as cohesive, adhesive, or mixed. The force needed to cause bond failures was recorded. Peel strength (N/mm) was determined [8]. After the peel strength test, rupture types were evaluated under a fluorescent light, and categorized into adhesive, cohesive, or mixed. Adhesive failure refers to complete separation at the interface between the PEEK and silicone, cohesive failure refers to tearing within the silicone material, and mixed failure refers to both.

The data were statistically analyzed by using a statistical software program (IBM SPSS Statistics, v29.0; IBM Corp., New York, US). Mean values and standard deviations, median, minimum, maximum values, and interquartile ranges (IQRs) for both bond strength and the surface roughness were calculated. Kruskal Wallis test was used to compare differences among groups (α = 0.05). The data for the results are shown in Table 1 and Table 2.

## 3. Results

Table 1 shows the surface roughness values of the groups in this study. Silica-coated PEEK specimens showed the highest surface roughness values, which was statistically significant compared to those of control PEEK specimens (*p* < 0.05). There were no statistically significant differences among the groups of silica-coating, laser, sandpaper, or plasma-treated specimens (*p* > 0.05).

As seen in Table 2, there was statistical difference between the groups regarding the peel test (*p* ˂ 0.05). The control PEEK group showed the highest peel bond strength, where only primer was used as a surface treatment. However, there was no statistical difference between the control, sandpaper, or plasma etching groups. The nano-fiber laser group demonstrated the lowest bond strength, and this result was not found to be significantly different from the silica-coating and PMMA groups. However, there was a statistically significant difference between the nano-fiber laser group and the control, sandpaper, and plasma groups. The positive control group, consisting of PMMA specimens, had statistically lower bond strength than either the PEEK control, sandpaper or the plasma etching groups.

**Figure 3:**
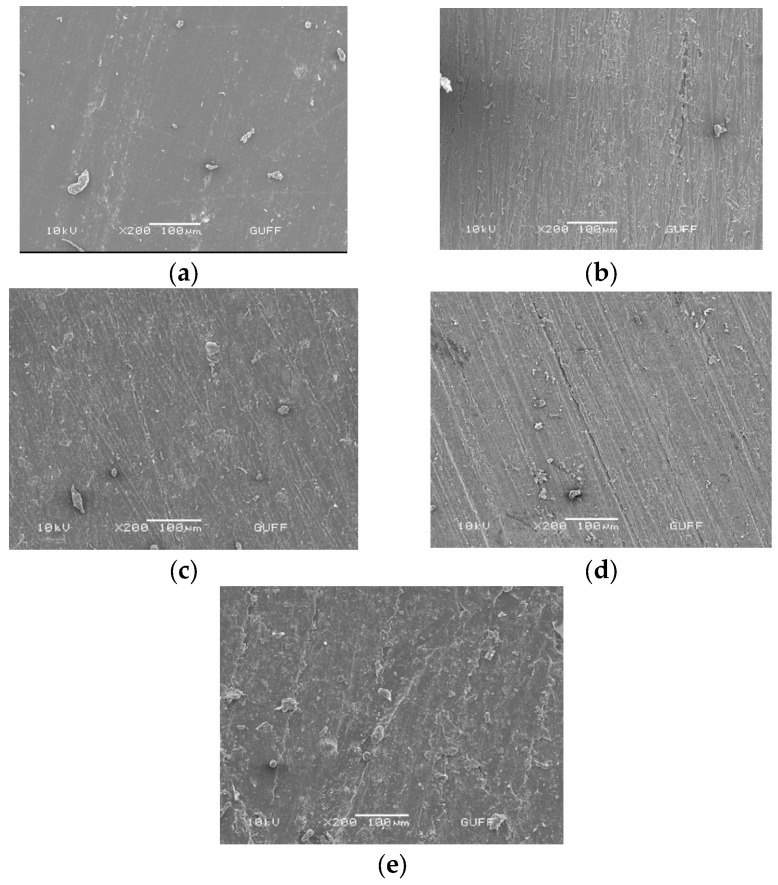
SEM images of control PEEK (**a**); sandpaper (**b**); oxygen plasma-treated (**c**); nano second fiber laser (**d**); and 30 µm Al_2_O_3_ silica-coating (**e**) specimens.

Figure 3a–e show the results of SEM surface analysis of the different study groups:

In SEM images, plasma-treated specimens exhibited horizontal directional scratches in their surface topography. The SEM images of nano fiber laser group specimens revealed noticeable irregular structures and cracks on the PEEK surface. Specimens in the silica-coating group showed similar surface topography to those in plasma-treated specimens. In contrast, relatively smooth surfaces were observed in the SEM analysis of the control PEEK specimens. Silica-coating specimens showed remarkable point surface changes of the Al_2_O_3_ particles remaining on the surface and from sandblasting. These observations provided valuable insights into the effects of different surface treatments on surface topography, which can inform the development of improved material surface properties.

**Figure 4:**
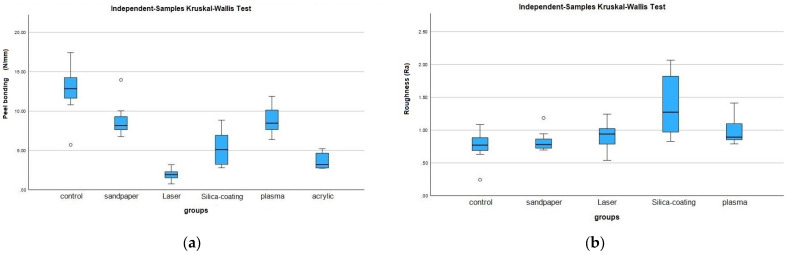
(**a**) Peel test results graphic in N/mm for the study groups. (**b**) Surface roughness values (Ra) for PEEK study groups.

In addition, Figure 4a,b show median values with IQRs for all groups in terms of peel test and surface roughness.

All the failure types were seen as adhesive type in this study.

## 4. Discussion

The hypotheses of this study were rejected as different surface treatments on PEEK affected the bond strength between PEEK and maxillofacial silicone differently. Furthermore, there was significant difference between study groups regarding the bond strength test.

To evaluate the bonding of maxillofacial silicones with resin materials, various test methods can be employed, such as the tensile test, shear test, and peel test [2,4]. The peel test is particularly suitable for simulating the horizontal component of detaching forces that occur when the patient removes the craniofacial implant-retained prosthesis [35]. For this reason, in our study, we used the 90° peel test to assess the bond strength.

Apart from modified PEEK, also known as BioHPP, with a nano-ceramic content of 20%, it has been reported that the pure PEEK material has a gray-white opaque color and that the PEEK surface should be coated with dental composite resin in order to meet aesthetic expectations in intraoral cavity [36,37,38]. In this regard, various surface treatments have been introduced for PEEK, including sandblasting, silica coating on PEEK surface [27,28,39], 98% sulfuric acid application [32,39], adhesive primer or resin applications [40,41], cold active gas plasma application [29,39], and different laser treatments [28,30].

Despite the emergence of high-power fiber laser systems with advanced features such as air cooling, nanoscale precision, and high-quality beams, their use in dental applications is limited. Nanofiber lasers can modify the material surface mechanically without causing excessive thermal change, unlike conventional laser systems that may create fractures and cracks. These lasers can generate surface topographies at nano- and micron-level scales [42,43]. In our study, SEM photographs showed that nanofiber laser treatment did not produce distinct surface changes visible at 100 μm magnification. Future studies with advanced imaging techniques could investigate the nanoscale changes made by the laser and analyze their homogeneity and effects on PEEK surface properties. Due to the high surface strength of PEEK, only a few surface treatments are effective in providing sufficient surface roughness [26,30,44,45]. Tsuka et al. [30] found that Nd:YVO4 laser treatment increased surface roughness on PEEK, providing optimal bonding between resin and PEEK through micromechanical locking. Laser systems using 2–3 W power have been recommended to avoid overheating defects on material surfaces when applied to resin materials [46,47,48]. The nanofiber laser used in this study had a power of 2 W, based on conventional laser systems in the literature. The surface roughness values generated by the nanofiber laser system were lower than the sandblasting group (*p* < 0.05), but there was no significant difference between the laser and other groups (*p* > 0.05). However, the bonding values of the nanofiber laser-treated specimens were significantly lower than the other groups (*p* < 0.05), suggesting that the nanofiber laser triggered surface degradation on the PEEK surface that disrupted the connection with silicone.

Plasma treatment has been reported to be an effective way to increase the surface energy of the polymer surface [49]. The type of plasma (argon plasma or oxygen plasma) can affect the wettability of the polymer surface. Soft non-thermal plasma, which involves oxygen plasma treatment, has been recognized for its ability to considerably increase hydrophilicity by generating multiple radicals, including excited oxygen molecules, OH radicals, atomic oxygen, superoxide anion radicals, and singlet oxygen without causing thermal damage or altering the surface roughness [50,51]. In our study, plasma treatment did not increase the surface roughness of PEEK specimens as compared with PEEK control group specimens (*p* > 0.05). In addition, plasma treatment did not show a damaging effect on SEM images of the PEEK specimens. Moreover, the bond strength of the plasma-treated specimens was observed to be lower in comparison to the control PEEK specimens, which demonstrated the highest bond strength within the study. However, it is worth noting that the observed difference in bond strength between the two groups was not found to be statistically significant (*p* > 0.05). These results suggest that the application of oxygen plasma increases the surface energy of PEEK, resulting in a stronger bond between silicone and PEEK. However, the increase in surface energy in the plasma group is not as substantial as that of the control group since the bond strength values were lower than those of the control group.

Sandblasting alone is not sufficient for bonding PEEK and resin without a primer [52]. Other studies have found that both silica coating and sandblasting improve bond strength through surface modification [26,28]. In our study, silica coating resulted in lower bond strength values than sandpaper, plasma, and control groups (*p* < 0.05), indicating that it may not enhance bond strength between PEEK and silicone elastomer. On the other hand, the silica-coating group showed higher surface roughness values compared to the plasma group, but this difference was not statistically significant (*p* > 0.05). In contrast, the bond strength values of the plasma group were higher than those of the silica-coating group (*p* < 0.05). The observed significant increase in surface roughness of the silica-coated PEEK specimens presented in this statement could be an important indication of the coating’s effectiveness in altering the surface properties of PEEK. However, it is important to consider the influence of other factors such as coating thickness and adhesion to the substrate, which could also affect the coating’s overall performance.

In our study, the control group exhibited the highest bond strength values, which were higher than the laser, sandblasting, and plasma groups (*p* < 0.05). However, the surface roughness values of the control group were lower than those of the sandblasting and plasma groups (*p* < 0.05). These results suggest that platinum primers can chemically enhance the bond between silicone and PEEK. It is noteworthy that the bond strength values of the plasma and silica-coating groups, which were expected to increase with the surface roughness generated by mechanical adhesion, were lower than those of the control group with platinum primer. This finding supports the notion that the PEEK–silicone connection is associated with chemical bonding rather than mechanical adhesion.

Maxillofacial silicone elastomers are dimethyl siloxane polymers, while acrylic resins are methyl methacrylate-based polymers [7]. Bonding problems between silicone and acrylic caused by different chemical structures between the two materials can often occur. The bond strength between acrylic substructure and silicone can be improved by using primer on the acrylic structure [3,8,9]. Furthermore, studies have shown that there is a need to apply an adhesive or primer for optimal bonding between the silicone/resin interface [3,4,8,22]. The bond strength between hard acrylic substructure or polyurethane substrate and silicone elastomer may be affected by the type of substrate and primer content. According to this, solvent-based primers are defined as a surface pretreatment method that can modify the polymer surface and result in stronger bonding [53]. The use of a primer to increase surface energy and wettability is a common practice in achieving strong bonding between different materials [54,55]. Organocylane-based primers are often used on silicone surfaces to improve bonding and chemical compatibility [54]. The organocylamine-based primers containing vinylsilan are typically used for bonding platinum-based silicone elastomers. These primers contain solvents such as water, alcohol, or organic solvents such as xylene, toluene, or naptha solution [55]. Studies have demonstrated that primers containing solvents such as ethyl acetate or methylene chloride can result in strong bonding between silicone and polyurethane substrates [3,8,56]. Control PEEK specimens treated with platinum primer tended to show the highest bond strength in this study. The platinum primer used in the study may have improved the wettability and surface energy of PEEK without causing any degradation.

The use of 3D printing technology has revolutionized many industries, including the medical field. This technology, also known as rapid prototyping or additive manufacturing, has the potential to reduce material costs and provide faster solutions for clinical and laboratory applications [57,58,59]. PEEK is a material that has gained interest in the medical and dental fields due to its unique properties. Its lightweight and polishable nature, as well as its color stability and low water absorption, make it an ideal material for maxillary obturators, which are usually made of PMMA resin [60,61,62]. PEEK’s ability to resist polymerization shrinkage during the manufacturing process also makes it a good candidate for maxillofacial prosthetic treatment options, particularly as an infrastructure material [45]. Overall, 3D printing technology combined with PEEK material has great potential for improving and advancing the field of maxillofacial prosthetics [60,61] Future studies should include larger specimen sizes and investigate the combined effects of mechanical and chemical processes on the bond strength between PEEK and silicone.

## 5. Conclusions

Within the limitations of this study, the following conclusions could be drawn:

1. This study demonstrated that applying a platinum primer to PEEK structures resulted in a favorable bonding between platinum silicone elastomer and PEEK.
2. Surface treatments on PEEK did not affect the bonding between PEEK and silicone. The obtained bond strength values revealed that PEEK may be a preferred supporting material over PMMA frameworks for fabricating implant-supported maxillofacial silicone elastomers. Notably, the control PMMA specimens exhibited significantly lower bond strength compared to the control PEEK specimens.
3. The strong bonding observed between silicone and PEEK has practical implications in the development of maxillofacial silicone prostheses, benefiting both patients and clinicians.
4. Future studies with larger specimen sizes should focus on investigating the bonding of PEEK frameworks with implant-supporting parts in such prostheses, further advancing our understanding in this area.
5. Additionally, future studies could explore a wider range of surface treatment parameters and use larger specimen sizes to provide a more comprehensive understanding of the bonding characteristics between PEEK and silicone in maxillofacial silicone prostheses.

## Figures and Tables

**Table 1:** Surface roughness values (Ra).

Groups	Median	IQR
Control PEEK	0.77 ^a^	0.63–0.88
Sandpaper	0.78 ^ab^	7.10–10.03
Laser	0.94 ^ab^	0.64–1.04
Silica coating	1.27 ^b^	0.87–2.01
Plasma	0.89 ^ab^	0.81–1.17
*p*	<0.001	

Different superscript letters indicate the statistical difference between groups in each column.

**Table 2:** Peel test results of the groups (N/mm).

Groups	Median	IQR
Control PEEK	12.85 ^a^	10.80–14.84
Sandpaper	8.15 ^a^	7.10–10.03
Laser	1.93 ^b^	1.12–2.67
Silica coating	5.11 ^b^	3.08–7.93
Plasma	8.47 ^a^	7.53–10.34
PMMA	3.21 ^b^	2.76–4.79
***p***	<0.001	

Different superscript letters indicate the statistical difference between groups in each column.

## Data Availability

Not applicable.
